# A Partition Function Approximation Using Elementary Symmetric Functions

**DOI:** 10.1371/journal.pone.0051352

**Published:** 2012-12-12

**Authors:** Ramu Anandakrishnan

**Affiliations:** Department of Computer Science, Virginia Tech, Blacksburg, Virginia, United States of America; University of Nottingham, United Kingdom

## Abstract

In statistical mechanics, the canonical partition function 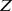 can be used to compute equilibrium properties of a physical system. Calculating 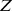 however, is in general computationally intractable, since the computation scales exponentially with the number of particles 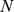 in the system. A commonly used method for approximating equilibrium properties, is the Monte Carlo (MC) method. For some problems the MC method converges slowly, requiring a very large number of MC steps. For such problems the computational cost of the Monte Carlo method can be prohibitive. Presented here is a deterministic algorithm – the direct interaction algorithm (DIA) – for approximating the canonical partition function 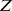 in 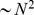 operations. The DIA approximates the partition function as a combinatorial sum of products known as elementary symmetric functions (ESFs), which can be computed in 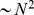 operations. The DIA was used to compute equilibrium properties for the isotropic 2D Ising model, and the accuracy of the DIA was compared to that of the basic Metropolis Monte Carlo method. Our results show that the DIA may be a practical alternative for some problems where the Monte Carlo method converge slowly, and computational speed is a critical constraint, such as for very large systems or web-based applications.

## Introduction

In statistical mechanics, the partition function 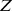, for a canonical ensemble of particles is given by
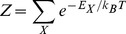
(1)where 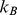 is the Boltzmann constant, 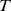 is the temperature, 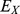 is the energy of microstate 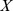, and the sum is over all accessible microstates [1]. The partition function can be used to calculate macroscopic thermodynamic properties of systems in equilibrium [2]. For applications where energy is a function of non-uniform interactions between particles, [3]–[7] such as the 3D Ising model for magnetic phase transition, the calculation of the partition function 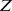 in Eq. (1), has been shown to be computationally intractable, i.e. NP complete [8]–[11]. One of the most commonly used methods for approximating equilibrium properties, is the Monte Carlo (MC) method, which scales as 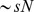, where 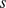 is the number of Monte Carlo steps and 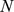 is the number of particles in the system. However, for problems involving systems with long-range correlation the MC method converges slowly [12], [13], and therefore may be impractical for some applications.

**Figure 1:**
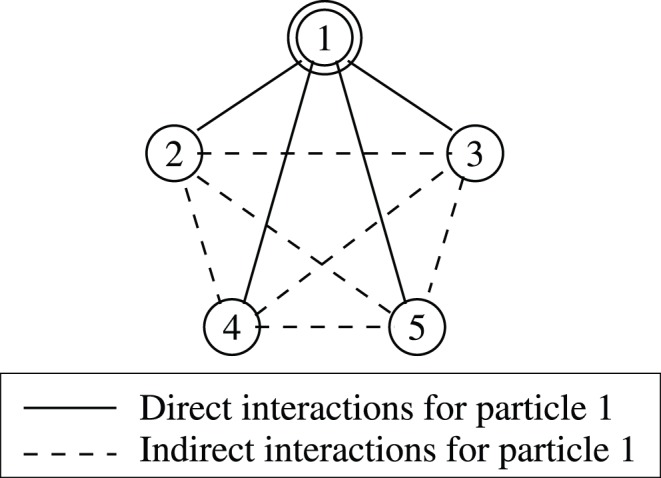
Direct and indirect interactions. For the five particle system shown here, with particle 1 as the selected particle, the direct interactions are shown as solid lines, and indirect interactions as dotted lines.

Presented here is a deterministic approximation – direct interaction algorithm (DIA) – for computing the partition function 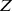 in Eq. (1). The DIA scales as 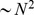 in the number of particles. For a selected particle, the DIA defines direct interactions as pairwise interactions involving the selected particle, and indirect interactions as pairwise interactions that do not involve the selected particle, as illustrated in Fig. 1. The DIA computes the exact contribution of direct interactions to the partition function, while using an average value for indirect interactions. In the hypothetical case where all the indirect interactions are equal, the partition function calculated by the DIA is exact to within numerical precision.

The DIA was applied to the computation of thermal average magnetization, internal energy and heat capacity for the isotropic 2D Ising model with no external field, for system size ranging from 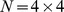 to 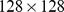, and the dimensionless temperature range of 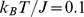 to 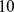, where 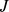 is the interaction potential between neighboring sites in the 2D Ising model. The accuracy of the DIA was compared to that of the commonly used basic (single-flip without biased sampling) Metropolis Monte Carlo (MC) method. Accuracy was measured as the root mean square (RMS) error relative to the exact computation.

Many other methods have been developed for approximating thermodynamic properties using the principles of statistical mechanics, though none are as widely used as the MC method, for practical problems. Most likely, this is due to the higher computational costs required for these other methods to achieve comparable levels of accuracy. For example, for systems involving non-uniform long range interaction, preliminary results (not included here) indicate that two such methods, the Wang Landau [14], [15] and the cluster Monte Carlo [16], [17] methods, can take two or more orders of magnitude longer to converge to accuracy levels comparable to that of the Monte Carlo method.

The accuracy of the MC method depends on the number of MC iterations used in the computation. In general, if the MC method is run for a sufficiently large number of iterations, it will converge to the correct solution within some desired level of accuracy. However, for very large problems and/or web-based applications computation time can be a critical constraint, such as for the computation of protonation states in biomolecules [18], [19]. To determine if the DIA may be a practical alternative to the MC method for such applications, we chose the number of MC iterations to be such that its computation time is at least 10 times longer than that of the DIA. For the 2D Ising systems considered here, the MC method converges slowly for temperature around and below the critical temperature of 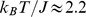. For MC runs with computation times that are 10 times longer than for the DIA, thermal average magnetization and heat capacity computed by the DIA are on average more accurate than those computed by the MC, while internal energy is more accurate for the MC method. Thus, for some problems where the MC method converges slowly and computation time is a critical constraint, the DIA may be a practical alternative.

The remainder of this paper is organized as follows. The *Methods* section provides a detailed derivation of the DIA. The accuracy of the DIA for the 2D Ising model are discussed in the *Results and Discussion* section. Finally, the method and results are summarized in the *Conclusions*.

## Methods

The direct interaction algorithm (DIA) assumes that there are only two possible states for each particle. This is true for many problems. For example, in the electrostatics of biomolecules, each ionizable amino acid can have absolute charge of 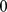 (neutral) or 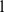 (charged), and in the 3D Ising model, each particle can have a spin of 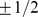. Moreover, systems with more than two states can be mapped to a two state system by replacing each particle with a set of two state “pseudo” particles [20]. Without loss of generality we also assume that the values of the two possible states are 0 and 1, because any two state problem can be mapped to an equivalent problem with states {0,1} [21].

For a selected site 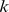, the DIA defines direct interactions as pairwise interactions 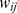 that involve the selected site, i.e. 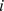 or 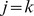. Indirect interactions are defined as pairwise interactions 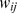 that do not involve the selected site, i.e. 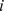 and 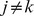. See Fig. 1. A previous study [21] suggested that, for some problems, direct interactions may be more important than indirect interactions for the computation of the partition function. Based on this finding, the DIA computes the contribution of direct interactions exactly, while using an average value for indirect interactions. By design the partition function calculated by the DIA is exact when all indirect interactions are equal. The algorithms and computations used by the DIA are derived below.

The derivation of the DIA consists of three steps. (1) The partition function 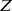 in Eq. (1) is reformulated to partition all possible microstates into subsets with the same number of indirect interactions. (2) The indirect interactions are approximated by an average value. (3) The contribution of direct interaction to the partition function is represented as a combinatorial sum of products known as elementary symmetric functions, which are computed in 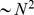 operations using the binary split-merge algorithm described below.

**Figure 2:**
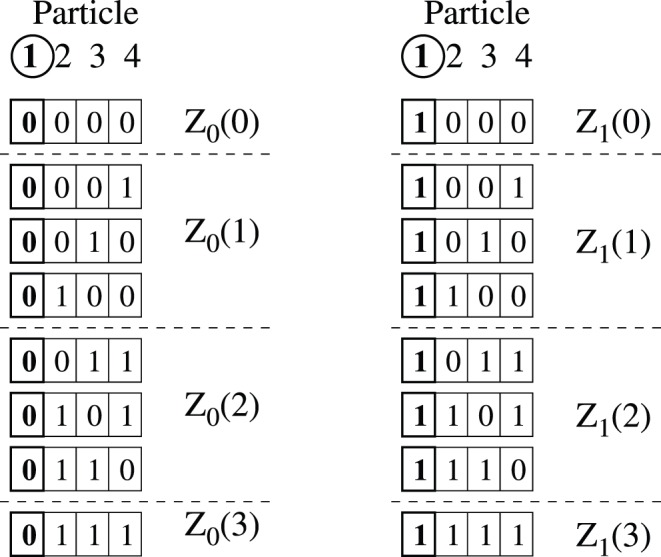
Partitioning of microstates. For a 4 particle system, the set of all possible 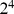 microstates are partitioned into subsets of states as follows. With particle 1 as the selected particles, the microstates are first partitioned into two subsets, one with particle 1 in state 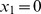 and another with 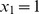, with contributions to the partition function corresponding to 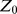 and 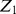 respectively (Eq. (3)). Each of these subsets are further partitioned into subsets with the same number of particles, 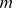, in state 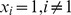, with contributions to the partition function corresponding to 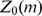 and 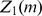 in Eq. (10) and (11) respectively.

### Step 1: Partition Microstates into Subsets with the Same Number of Indirect Interactions

The partition function in Eq. (1) involves the summation over all 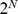 possible microstates 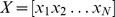, where 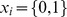 is the state of particle 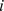. This summation can be subdivided into the sum over two subsets of microstates. One subset in which the state of the selected particle 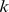, 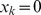, and another subset in which 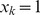, as illustrated in Fig. 2, i.e.
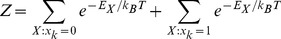
(2)

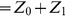
(3)where 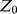 and 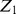 are the contributions to the partition function due to microstates with 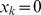 and 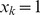 respectively.

Each of these subsets of microstates can be further subdivided into 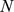 subsets each, such that each subset has the same number of particles in state 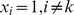, as illustrated in Fig. 2, i.e.
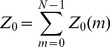
(4)

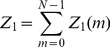
(5)where 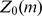 and 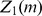 represent the contribution to the partition function due to microstates with 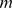 particles in state 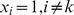, and with the particle 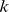 being in state 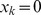 and 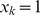 respectively.

The contribution to the partition function due to each of these subsets of microstates can now be represented as the product of contributions due to direct interactions and indirect interaction, i.e.
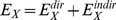
(6)

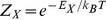
(7)

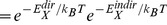
(8)

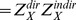
(9)

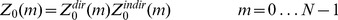
(10)

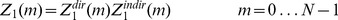
(11)where 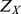 is the contribution to the partition function due to state 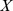, and the superscript “
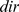
” and “
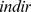
” represent the contributions due to direct and indirect interactions respectively.

### Step 2: Approximate the Contribution Due to Indirect Interactions

The contribution to the partition function due to indirect interactions in Eq. (10) and (11) can be approximated as follows
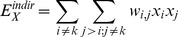
(12)

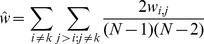
(13)

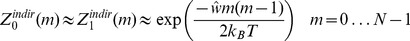
(14)where 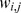 is the interaction between particles 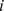 and 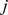, and 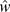 is the average indirect interaction. The factor 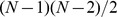 in Eq. (13) is the total number of indirect interactions, and the factor 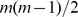 in Eq. (14) is the number of indirect interactions when 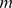 particles are in state 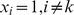.

### Step 3: Calculate the Exact Contribution Due to Direct Interactions

The contribution to the partition function due to direct interactions 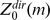 and 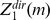 can be represented by the combinatorial sum of products known as elementary symmetric functions 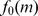 and 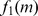, as follows
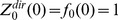
(15)

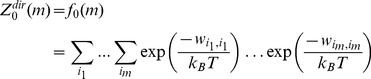
(16)

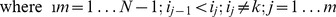


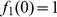
(17)

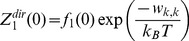
(18)

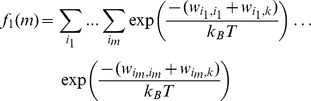
(19)

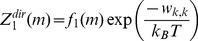
(20)

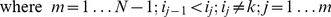



In Eq. (15) and (16) the selected particle 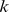 is in state 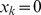, and in Eq. (17)–(20) 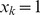. When 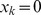, there is no direct interaction between 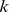 and any of the other particles (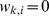), and the contribution to 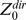 only consists of the self interaction terms 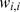. When 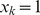 the contribution to 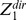 consists of the self interaction terms 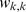 and 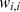, and the direct interaction term 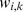.

Although the summations in Eq. (16) and (19) consist of 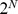 terms, where each term represents the contribution due to one of the 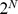 microstates of the system, the elementary symmetric functions themselves can be computed recursively in 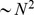 operations using the Newton’s identity generating function [22], [23]. However, the Newton’s identity generating function can suffer from large numerical errors due to catastrophic cancellations, the loss of precision in computations involving small differences between very large number. For example, for a 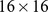 2D Ising model, as described below, the partition function 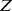 calculated using Newton’s identity is 2.25, whereas the correct value, without the numerical error is 2.40. The above computations were carried out with 64-bit double precision arithmetic.

Presented here is an alternate algorithm – the binary split-merge algorithm – that avoids the catastrophic cancellations inherent in the Newton’s identity calculations. The binary split-merge algorithm is based on the following theorem [24].

#### Theorem 1

*If a system is partitioned into two subsystems 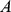 and 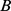, then the elementary symmetric function 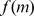 for the combined system can be calculated from the elementary symmetric functions 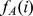 and 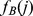, for 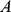 and 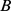 respectively, as follows.*

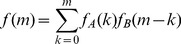
(21)


##### Proof

1. Let 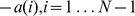 be the roots of a polynomial 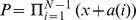, where 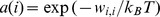 which are the individual terms in the ESF 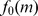 (Eq. (16)).
2. Then each elementary symmetric function 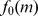 in Eq. (16) is the coefficient of 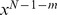 in the polynomial 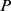.
3. Let the subsystem 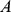 consist of particles 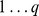, and the subsystem 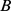 of particles 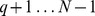.
4. Then 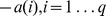 are the roots of the polynomial 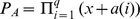 with the ESF for 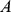, with 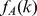 being the coefficients of 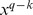 in the polynomial 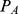. And 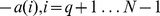 are the roots of the polynomial 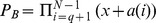 with the ESF for 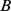, with 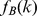 being the coefficients of 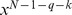 in the polynomial 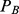.
5. Also, 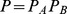 since 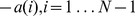 are the roots of the polynomials 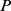 and 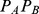.
6. Therefore, 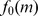 the coefficients of 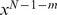 in 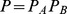 are

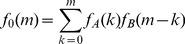
(22)The above proof is for the ESF 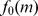. The same proof applies to the ESF 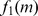 in Eq. (19) with 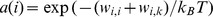.

**Figure 3:**
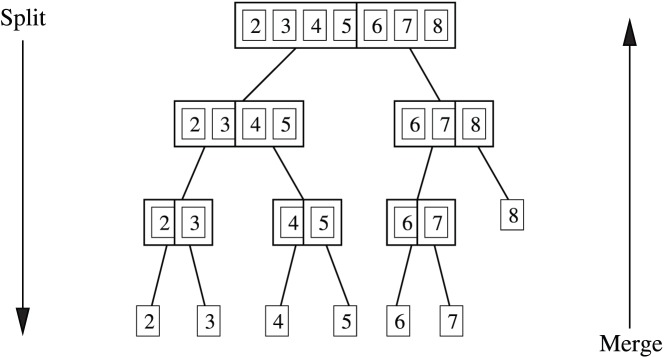
Split-merge algorithm. Consider an 8-particle system, with particle 1 being the selected particle. First, the split-merge algorithm recursively separates the particles, other than the selected particle, into a hierarchical binary tree. Next, the elementary symmetric function (ESF) for the leaf nodes, which consist of a single particle, are calculated. The ESF for all the other nodes, are then computed by recursively, starting from the bottom, merging the ESF from the two branches for each node using Eq. (21).

The binary split-merge algorithm consists of two stages, as illustrated in Fig. 3. First, in the split stage, the particles are recursively split into a binary tree where each node with 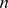 particles has two branches with ⌊*n*/2⌋and *n*−⌊*n*/2⌋;particles in each. The elementary symmetric function values, for the leaf nodes with only one particle 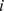, are 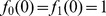, 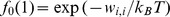, and 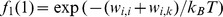. Then, in the merge stage, the elementary symmetric function from the two branches of a node are recursively combined, starting from the bottom, using Eq. (21).

An additional benefit of the binary split-merge algorithm is that it can be used to reduce the error in the indirect interaction approximation in Eq. (14). Instead of using the same average value 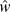 for all indirect interactions, one can include in the partition function approximations (Eq. (10) and (11)) an average indirect interaction value in each merge step that is based only on the indirect interactions between the subset of particles being merged, as follows. Let 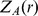 be the contribution to the partition function, including direct and indirect interaction, for the subset 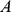 (Eq. (21)) with 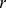 particles in state 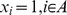. And let 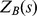 be the contribution to the partition function, for the subset 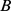 with 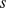 particles in state 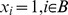. Then, applying the same principle used in Eq. (21), the contribution to the partition function for the combined system 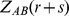 can be approximated as:
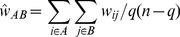
(23)

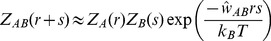
(24)where 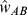 is the average indirect interaction between the subsets 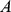 and 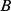 with 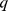 and 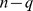 particles respectively, and 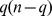 are the number of indirect interactions across the two subsets. If the indirect interactions between 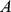 and 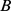 are all equal, then Eq. (24) is exact.

It may be possible to increase the accuracy of the DIA by selecting multiple particles, for determining direct and indirect interactions. Any interactions involving any of the selected particles would be treated exactly (direct interactions), while an average value would be used for interactions not involving any of the selected particles (indirect interactions). Since fewer interactions are approximated, this modification should improve accuracy. However, the modification would require reformulation of the equations developed above, and would increase computational cost. Such a modification to the DIA has not been explored in this study.

### 2D Ising Model

The 2D Ising model of ferromagnetism was selected to test the accuracy of the DIA. It is one of the most thoroughly investigated system in statistical mechanics [25], [26]. Despite its simplicity the 2D Ising model exhibits many of the thermodynamic properties, such as spontaneous magnetization, of real, and much more complex, systems. Thus one can gain insights into these more complex systems through the study of the simpler 2D Ising model. Moreover, there exists an exact solution for the isotropic 2D ising model with periodic boundary condition and no external field. The exact solution by Onsager [27], was generalized by Kaufman [28], and implemented by Beale [29], [30]. This open source implementation computes exact values for thermal average magnetization 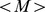, internal energy 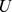, and heat capacity 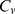, for a given system size and temperature range.

The exact values for 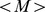, 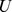, and 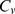, for the 2D Ising model described below, provided the baseline for evaluating the accuracy of the DIA. The accuracy of the DIA was also compared to that of the basic (single-flip without biased sampling) Metropolis Monte Carlo (MC) method. The MC method includes a thermalization phase, prior to sampling data for estimating the above thermodynamic properties. The same number of steps were used for the thermalization phase as for the sampling phase.

The Ising model used for testing the DIA is an isotropic 2D model with nearest neighbor interaction potential 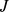, on an 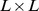 lattice, with periodic boundary conditions and no external field. Each vertex of the lattice can have a spin of 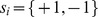. The energy 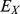 of this system in state 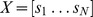 is given by
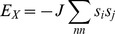
(25)where the number of lattice sites 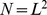 and the sum is over pairs of nearest neighbors (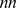) only. For the purpose of this analysis, a value of 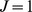 is used. The thermal average magnetization 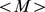, internal energy 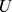, and heat capacity 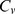 are defined as follows:
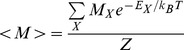
(26)

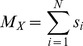
(27)

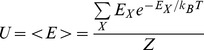
(28)

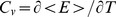
(29)

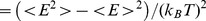
(30)

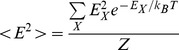
(31)where 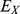 is the energy of state 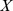, 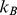 is the Boltzmann constant, 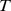 is the temperature, and 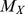 is the total magnetization (spin) of state 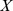.

The spin states 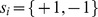 in the 2D Ising model can be mapped to 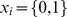 in the DIA as follows:
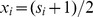
(32)

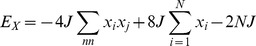
(33)

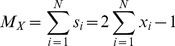
(34)


For the DIA, 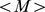, 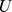, and 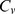, with the mapping from 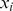 back to 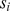, are computed as follows:
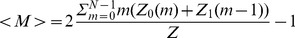
(35)

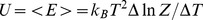
(36)

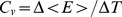
(37)where 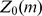 and 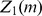 are as defined earlier by Eq. (10) and (11). 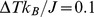 is used to numerically compute the derivatives in Eq. (36) and (37).

The 2D Ising model described above, was chosen here for assessing the accuracy of the DIA because an exact solution is available for this system. However, many practical problems do not have a tractable exact solution. To evaluate the effectiveness of the DIA for such problems, it may be possible to use the Monte Carlo method to define a baseline for comparison. If the MC method is run for a sufficiently large number of MC steps, the results should converge to the correct value. Therefore, for some problems the results from a MC run with a very large number of MC steps may serve as a baseline for comparison.

## Results and Discussion

To make the quantities in the following analysis dimensionless, temperature 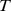 is divided by 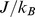, internal energy 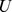 is divided by 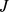, and heat capacity 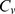 is divided by 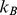, where 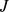 is the nearest neighbor interaction potential and 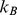 is the Boltzmann constant.

The DIA was used to compute thermodynamic properties for 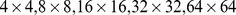 and 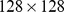 2D Ising systems described above. Accuracy of the DIA was compared to that of the basic Metropolis Monte Carlo (MC) method. Accuracy was measured as the RMS error relative to the exact computation for the dimensionless temperature range 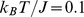 to 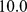 in increments of 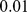. Computation time was measured by CPU time (all testing was performed on a workstation with a dual core Intel pentium 4, 3.2 GHz processor).

A goal of the following analysis is to determine if and when the DIA may be a practical alternative to the MC method, specifically for applications with computation time constraints, such as web services for computing protonation states in biomolecules [18], [19]. Therefore, for the results shown below the number of Monte Carlo steps for the MC method is chosen such that MC computation time is at least 10 times longer than that of the DIA. See Table 1. Note that for these computation times, the MC method may not have sufficiently converged. Therefore, the following comparisons are only meaningful in the context of applications where computation time is a critical consideration. Where computation time is not a critical constraint, in general the MC method can be run long enough to achieve a desired level of accuracy.

**Table 1:** Number of Monte Carlo (MC) steps.

Size	4×4	8×8	16×16	32×32	64×64	128×128
DIA CPU sec.	0.0012	0.0019	0.0120	0.1618	2.4834	39.0148
MC CPU sec.	0.0144	0.0196	0.1460	1.6276	27.3846	417.5865
MC steps	1.5×10^4^	1.0×10^4^	2.0×10^4^	2.0×10^4^	6.0×10^4^	2.0×10^5^

The number of MC steps is chosen such that the computation time (CPU time) for the MC method is at least 10 times the computation time for the direct interaction algorithm (DIA).

**Figure 4:**
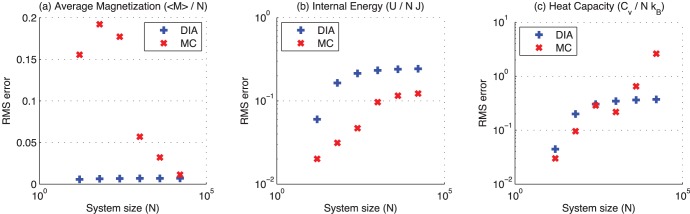
Accuracy as a function of system size. Accuracy for the direct interaction algorithm (DIA) and the Metropolis Monte Carlo (MC) method as a function of system size 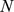 (log-log scale). Accuracy is calculated as the RMS error relative to the exact value. The number of steps is chosen such that the computation time for the MC method is at least 10 times the computation time for the DIA (Table 1).

Table 2 summarizes the overall accuracy (RMS error) for the DIA and the MC method, and Figure 4 shows the accuracy for the two methods as a function of system size. The MC method, after computation times that are 10 times longer than that of the DIA, has not sufficiently converged. As a result thermal average magnetization 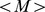 and heat capacity 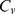 calculated by the MC method are less accurate on average than for the DIA. Internal energy 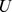 calculated by the MC method is however more accurate than the DIA for these computation times.

**Table 2:** Accuracy comparison.

	RMS error	
	DIA	MC
Average Magnetization (<*M *>/*N*)	0.0066	0.1270
Internal Energy (*U*/*NJ*)	0.2031	0.0829
Heat Capacity (*C_v _/ NK_B_*)	0.2952	1.1098

Accuracy for the direct interaction algorithm (DIA) and the Metropolis Monte Carlo (MC) method. Accuracy is calculated as the RMS error relative to the exact value. The number of steps is chosen such that the computation time for the MC method is at least 10 times the computation time for the DIA (Table 1).

**Figure 5:**
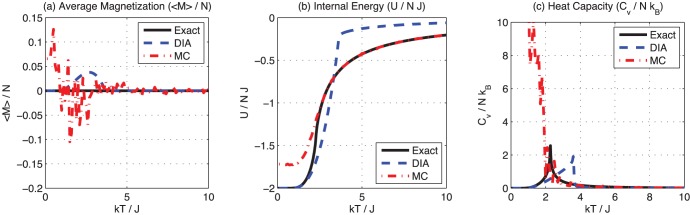
Calculated values for the 128×128 2D Ising system. (a) Thermal average magnetization 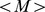, (b) internal energy 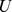 and (c) heat capacity 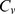, as a function of dimensionless temperature 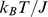 for the 128×128 2D Ising system, as calculated by the direct interaction algorithm (DIA), the Metropolis Monte Carlo (MC) method, and the exact solution. The number of steps for the MC method was chosen such that the MC computation time is at least 10 times the computation time for the DIA. Connecting lines are shown to guide the eye.

To better understand why the DIA may be more accurate in some cases, consider the values of these three thermodynamic properties, for the DIA and MC methods compared to the exact computation, as a function of temperature (Figure 5).

As expected, the MC method is less accurate around and below the critical temperature of 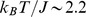, where long range correlation becomes important and where the MC method converges slowly. At higher temperatures, where there is little long range correlation, the MC method converges more rapidly. Note that techniques have been developed, such as clustering and umbrella sampling, that can improve the accuracy of the Monte Carlo method in the critical and low temperature regions for some problems.

As seen in Figure 5, accuracy of the DIA depends on the temperature and the thermodynamic property of interest. Let us take a look at accuracy of the DIA for each thermodynamic property – thermal average magnetization 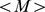, internal energy 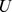, and heat capacity 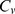 – in each of the three temperature regions: the low temperature region where 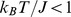, the high temperature region where 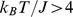, and the critical temperature region where 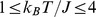. In the low temperature region the partition function 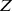 is dominated by the two low energy states where the spins are aligned, all 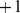 or all 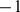. For these states, the energy 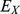 calculated using the average indirect interaction potential 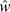, Eq. (13), is the same as the exact value. Therefore the DIA is quite accurate in the low temperature region for all three thermodynamic properties. In the high temperature region, the value of 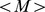 in Eq. (26) is dominated by the multiplicity for different spin states. Since the DIA uses the exact value for the multiplicity of spin states in its computations, 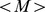 is quite accurate in the high temperature region. 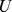 on the other hand also depends on energy 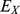, Eq. (28). In the high temperature region 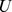 is dominated by the partition function 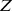 in Eq. (28). 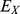 calculated using the average indirect interaction potential 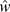 may be over or under estimated in this region, but since 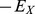 is in the exponent of 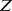, the underestimated terms in 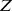 (which is in the denominator in Eq. (28) for 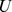) are dominant, and thus 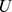 is overestimated, as seen in Figure 5(b). Heat capacity 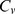 calculated by the DIA is however quite accurate in the high temperature region, Figure 5(c). The DIA uses the derivative of 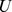 to compute 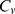, Eq. (37). Although DIA overstates 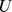, the value of 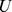 converges to the exact value as 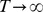, at close to the same rate as the exact computation. As a result the DIA closely approximates the correct value for 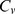 in the high temperature region. The DIA, in some sense being a mean-field like approximation, is not very accurate around the critical temperature, for all three thermodynamic properties. In this region, the accuracy of both energy 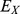 and the partition function 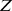 become important, and therefore the accuracy of the DIA is highly sensitive to any difference between the correct value for the indirect interaction potential 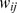 and the average value 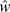 used by the DIA.

The above results suggest that for some problems where the MC method converges slowly, and where computation time is a critical constraint, the DIA may be a practical alternative.

### Conclusions

In statistical mechanics, the partition function 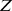 can be used to compute equilibrium thermodynamic properties of a system. However, the computation of the partition function scales exponentially in the number of particles 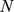, and is therefore in general intractable for systems with 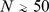. Therefore, most practical problems, the Monte Carlo (MC) method is commonly used to approximate the values of equilibrium thermodynamic properties. However for some problems, such as those with long range order, the MC method converges slowly and may be impractical for applications where computation time is a critical constraint, such as for large systems and/or web-based applications.

The direct interaction algorithm (DIA) presented here, approximates the partition function in 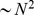 operations. For a selected particle, the DIA computes the contribution to the partition function due to direct interactions (interactions involving the selected particle) exactly, while using an average value for indirect interactions (interactions not involving the selected particle). Even with this approximation, the exact computation of the contribution due to direct interaction involves the calculation of the so called elementary symmetric functions, where the number of terms grow exponentially with 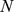. The Newton’s identity generating function can be used to recursively calculate these elementary symmetric functions in 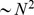 operations. However, the Newton’s identity generating function can result in large numerical errors due to catastrophic cancellations. A binary split-merge algorithm was developed to compute the elementary symmetric function without these numerical errors. This algorithm first partitions the system into a hierarchical binary tree where the root node contains the entire system and each leaf node contains a single particle. Then, starting with the leaf node, the algorithm recursively merges the elementary symmetric function for the two child nodes to compute the elementary symmetric function for the parent node. The software implementation of the DIA can be downloaded from http://sourceforge.net/projects/isingdia/files/.

The direct interaction algorithm (DIA) was used to calculate thermal average magnetization 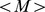, internal energy 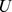, and heat capacity 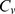 for the isotropic 2D Ising model with periodic boundary condition, no external field, and systems ranging in size from 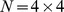 to 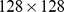. Accuracy of the DIA was compared to that of the basic Metropolis Monte Carlo (MC) method, where accuracy was measured as RMS error relative to the exact computation.

Where computation time is not a critical constraint, in general the MC method can be run long enough to achieve a desired level of accuracy. There are however many applications where computation time is a critical constraint, such as for very large systems or web-based applications. To determine the potential usefulness of the DIA for such applications, we compared the DIA to the MC method with the number of MC steps chosen such that the computation times for the MC were at least 10 times longer than that of the DIA. The MC method may not converge sufficiently for these computation times, and therefore this comparison is only meaningful for applications where computation time is a critical consideration. Our results show that, for these computation times and the systems considered here, the DIA can be on average more accurate than the MC method, for the computation of thermal average magnetization 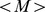 and heat capacity 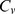. Thus the DIA may be a practical alternative for some problems.
